# Let-7a microRNA modulates caspase-3-dependent apoptosis in melanoma cells treated with dabrafenib and trametinib combination

**DOI:** 10.1007/s11845-025-03923-6

**Published:** 2025-03-10

**Authors:** Murat Keser, Harika Atmaca

**Affiliations:** 1 Department of Medical Oncology, Izmir Tepecik Education and Research Hospital, University of Health Sciences, Izmir, Türkiye; 2 Department of Biology, Faculty of Engineering and Natural Sciences, Celal Bayar University, 45140 Manisa, Türkiye

**Keywords:** Apoptosis, Dabrafenib, Let-7a, Melanoma, Synergism, Trametinib

## Abstract

**Background:**

Malignant melanoma is an aggressive tumor with high resistance to therapy. The emergence of RAS-driven secondary cancers and BRAF-inhibitor resistance has led to the development of combination therapies targeting both BRAF and MEK.

**Aims:**

This study explored the mechanisms underlying the synergistic effects of dabrafenib (DAB) and trametinib (TM) in drug-resistant A375 and RPMI 7951 melanoma cells.

**Methods:**

Cytotoxicity was assessed via MTT assay and combination effects were evaluated via combination index analysis. Apoptosis was analyzed by DNA fragmentation ELISA, while ectopic let-7a miRNA expression and inhibition were performed using lipofection. Gene expression levels were quantified by qRT-PCR, and protein expression was assessed via Western blot.

**Results:**

The combination of 0.7 μM DAB and 5.0 μM TM exhibited synergistic cytotoxicity by inhibiting the pERK1/2 signaling pathway and inducing MITF expression. This resulted in mitochondria-mediated apoptosis, characterized by a decrease in anti-apoptotic Bcl-2 and an increase in pro-apoptotic Bax, caspase-9, and caspase-3 levels. Additionally, Let-7a was identified as a crucial regulator of apoptosis sensitivity by targeting caspase-3, the key executor of apoptosis.

**Conclusions:**

These findings provide new insights into overcoming melanoma drug resistance through combined BRAF/MEK inhibition.

**Graphical abstract:**

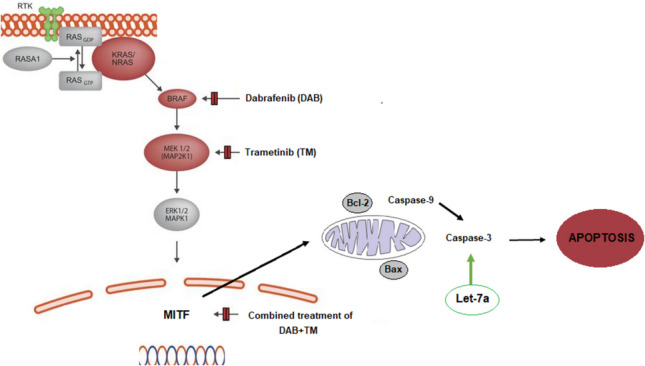

## Introduction

Malignant melanoma is one of the most aggressive tumors, and the average life expectancy in patients with metastatic melanoma is 6 to 10 months. With the development of targeted agents in recent years, the prognosis of melanoma patients has reached 80%, and survival rates have increased significantly. Although patients diagnosed at an early stage can be treated, the treatment of metastatic patients is quite difficult [1]. Since the treatment of advanced-stage patients is so difficult, there is an urgent need to investigate mutations in signal transduction pathways and to discover drugs targeting molecules in these pathways.

The BRAF V600E somatic mutation, discovered in 50% of melanomas, has contributed to the development of molecular targeted therapy for melanomas [2]. However, issues such as dose-related toxicity and intrinsic and acquired resistance remain major challenges in BRAF inhibitor therapy [3]. Resistance to chemotherapy, which is accepted as the core of cancer treatment, poses significant obstacles to the efficacy of the therapy. Changes in a variety of biomarkers, including epigenetic modifications, gene transmutation and/or amplification, and microRNA (miRNA) expression, are assumed to be responsible for the ability to adapt and develop chemotherapy resistance [4].

The main strategy for overcoming drug resistance phenomena is drug combination therapy in cancer therapy. Currently, more than 100 clinical trials are underway in combination with BRAF inhibitors. Among these combinations, combinations of mitogen-activated protein kinase (MEK) inhibitors and BRAF inhibitors have become the standard treatment for melanoma patients [5]. Vemurafenib is the first BRAF inhibitor that was approved by the FDA for use in advanced, irreversible, and metastatic melanomas. It has provided up to 80% response and increased survival rates compared to chemotherapy in metastatic melanoma patients [6]. Another BRAF inhibitor, dabrafenib (DAB), was approved by FDA in 2013. The randomized phase II study of DAB with TM significantly improved response rates (76 vs. 54%), prolonged progression-free survival (PFS; 9.4 vs. 5.8 months), and reduced toxicity in patients with BRAFV600-mutant metastatic melanoma [7]. Trametinib (TM) inhibits MEK1/2 by blocking its kinase activity, thereby preventing the phosphorylation of MEK by RAF [8]. Despite significant advances in clinical results and reports of prolonged responses, 50% of patients receiving DAB with TM have improvements within 9–10 months, and resistance continues to be an obstacle in enhancing patient outcomes. Possible resistance mechanisms may be overcome if the precise mechanisms of action of this combination are understood. Here, we investigated the underlying mechanism of synergistic DAB/TM combination in drug-resistant A375 and RPMI-7951 cells.

Phosphorylation of microphthalmia-associated transcription factor (MITF) at amino acid 73 by ERK has been reported to cause its degradation, which is a transcription factor unique for melanoma development and resistance (Truderung et al., 2021). It has a vital role in the signaling steps of survival pathways in resistant melanoma cells.

miRNAs are short (21–23 nucleotides), conserved, and non-coding RNA molecules that modulate various biological processes by binding to the 3′-untranslated region (3′-UTR) of target mRNAs and influencing the post-transcription of numerous genes. miRNAs are one of the important mechanisms that control the development of resistance, and many gene targets play a role in the development of melanoma.

In recent years, miRNAs have been recognized as key regulators of oncogenesis, influencing cell survival, proliferation, apoptosis, migration, and metastasis. Among them, miR-let-7a acts as a tumor suppressor in various cancers by targeting tumor-related genes [9–11]. It was shown that miR-let-7a was strongly expressed in melanocytes but significantly downregulated in malignant cells [12]. Since let-7 has tumor suppressive functions, it is an accepted potential therapeutic option for the treatment of advanced melanoma.

The study has confirmed that Let-7a plays a critical role in regulating the sensitivity of melanoma cells to apoptosis induced by combined therapy. This is achieved by targeting caspase-3, which is known as the main executor of apoptosis.

## Materials and methods

### Cell culture and reagents

A375 (ATCC# CRL-1619) and RPMI-7951 (ATCC# HTB-66) human melanoma cells were obtained from American Type Culture Collection (ATCC). RPMI-7951 cells were maintained in Eagle's Minimum Essential Medium (Sigma) with 10% fetal bovine serum, and A375 cells were cultured in Dulbecco's modified Eagle's medium (Sigma) with 10% fetal bovine serum (Sigma). Penicillin–streptomycin was added to the medium to prevent microbial and fungal contamination. Cells were maintained in incubators with 5% CO_2_ at 37 °C. The stock solutions of DAB (Selleckchem) and TM (Sigma) were prepared in dimethyl sulfoxide (DMSO). The DMSO concentrations in the wells were < 0.1% and not cytotoxic to the cells.

### XTT cell viability assay

Cells were seeded in a density of 10^4^ cells/well in 96-well plates. After 12 h of attachment, cells were treated with increasing concentrations of DAB or TM alone for 24, 48, and 72 h. After calculating the IC_50_ values of each drug via CalcuSyn software (Biosoft), DAB and TM were combined at varying concentrations for 72 h. After incubation periods, XTT solution (100 μL; Sigma) was added to each well and plates were incubated for an additional 4 h at 37 °C in a CO_2_ incubator. Optic densities were measured at 450 nm with a reference wavelength of 650 nm using a spectrophotometer (Tecan) [13]. Combination index (CI) values were calculated using CalcuSyn software. The CI was used to express additive effect (CI = 1), antagonism (CI > 1), synergism (CI < 1), and strong synergism (CI < 0.5) [14]. The most synergistic combination below the IC_50_ values, 0.7 µM DAB and 5.0 µM TM, was selected for further analyses.

## DNA fragmentation analysis

Apoptosis was evaluated by measuring DNA fragments using a commercial kit (Cellular DNA Fragmentation ELISA; Merck) according to the manufacturer’s instructions. Briefly, cells were plated on a 96-well microplate in a density of 10^5^ cells/well and exposed to drugs alone or in combination for 72 h. Cells were lysed via lysis buffer and cytoplasmic fractions (20 µL) were transferred to the streptavidin-coated ELISA plates. A mixture containing 80 µL anti-DNA and anti-histone solutions was added to each well and incubated for 2 h at 37 °C. After three washing steps with washing buffer, 100 µL diammonium salt solution was added to wells and optic densities were measured at 450 nm with 490 nm reference wavelength using a spectrophotometer (Tecan) [15].

### Ectopic let-7a miRNA expression and inhibition

Melanoma cells were either transfected with the let-7a precursor to up-regulate its expression or an anti-let-7a inhibitor to knock it down (Ambion). Melanoma cells were transfected with 25 nM of Let-7a precursor or anti-Let-7a inhibitor (Ambion, USA) using Lipofectamine 2000 (Invitrogen, USA), following the manufacturer’s protocol. The transfection duration was optimized at 24 h to ensure efficient uptake before subsequent drug treatments. To assess transfection efficiency, quantitative real-time PCR (qRT-PCR) was performed to measure Let-7a expression levels post-transfection, confirming significant upregulation in Let-7a precursor–transfected cells and downregulation in anti-Let-7a inhibitor–transfected cells. Additionally, a fluorescently labeled control siRNA was used in parallel experiments to visualize transfection efficiency under a fluorescence microscope, ensuring reproducibility and consistency across experimental conditions. As a control, cells were also transfected with commercially available precursor/inhibitor control (Ambion). Following transfection, the cells were either treated with drugs or lysed to extract protein or RNA.

### RNA isolation and cDNA synthesis

Total RNA was extracted from melanoma cells using miRNeasy kit (Qiagen), according to the manufacturer’s protocol. Additionally, on-column DNaseI digestion was conducted, and purification was done via an RNEasy MinElute (Qiagen). To determine let-7a expression, RNA underwent a reverse transcription reaction using the QuantMir RT Kit (System Biosciences, Mountain View, CA). RNA quantity and purity were measured using Nanodrop Spectrophotometer at 260 nm. RNA integrity was determined by running isolates on a 2% agarose gel and inspecting for distinct 18S, 28S, and tRNA bands, indicating a lack of degradation. cDNA synthesis was done using purified RNA via RT^2^ First Strand Kit (Qiagen) according to the manufacturer’s protocol.

### Quantitative reverse transcription PCR

Measurement of mRNA levels was conducted using Light Cycler 480 (Roche Applied Science, Mannheim, Germany). PCR master mix (2250 μL) was prepared using 2 × SuperArray RT^2^ qPCR Master Mix (Qiagen) and 102 μL of diluted cDNA. Standard cycling conditions (10 min at 95 °C, 15 s at 95 °C, 1 min at 60 °C for 40 cycles) for TaqMan reagents were used. For quality control, no reverse transcription control and no template control were performed. The primer sequences were as follows: caspase-3 (F) 5′-AGAACTGGACTGTGGCATTGA G-3′ and (R) 5′-GCT TGT CGG CAT ACT GTT TCA G-3′; GAPDH (sense) 5′-TGACTTCAACAGCGACACCCA-3′ and (reverse) 5′-CACCCTGTTGCTGTAGCCAAA-3′ (reverse); glyceraldehyde-3-phosphate dehydrogenase (GAPDH) was used as endogenous control gene. For each miRNA detection, the forward primer was the let-7a mature DNA sequence and the reverse primer was a 3′ universal primer provided by the QuantiMir RT Kit. Each replicate cycle threshold (C_T_) was normalized to the average C_T_ of two endogenous controls on a per-plate basis. The comparative C_T_ method was used to calculate the relative quantification of gene expression [16].

### Western blot analysis

Western blot analysis was performed as described before to detect successful transfection and subsequent expression of the MITF protein in melanoma cells [17]. Total protein isolation was performed using MPER Mammalian Protein Extraction Reagent (Thermo Fisher). Protein concentrations were measured via Bradford assay. Isolated proteins were separated on an SDS-PAGE and transferred to nitrocellulose membranes (Bio-Rad). Membranes were blocked using 5% nonfat dry milk containing 0.1% Tween 20. Membranes were treated with primer antibodies (pERK1/2, MITF, Bcl-2, Bax, Caspase-9, Casapase-3, β-actin) overnight at 4 °C and washed three times with TBST. Then, membranes were treated with secondary antibodies (1:1000 dilutions; Santa Cruz) for 2 h at RT and washed with TBST. Protein bands were visualized using UVP Imaging equipment and ImageJ software was used to quantify protein bands.

### Statistical analysis

Statistical analysis was done via GraphPad Prism (La Jolla, CA, USA). software. The data were analyzed by using a one-way analysis of variance test (ANOVA) followed by Dunnett’s test for multiple comparisons. Values with *p* < 0.05 were accepted as statistically significant.

## Results

### DAB in combination with TM inhibits cell viability synergistically in melanoma cells

To investigate the possible synergistic effect of DAB and TM combination on human A375 and RPMI 7951 melanoma cells, first, the IC_50_ values of DAB and TM were calculated. Cells were exposed to increasing concentrations of DAB or TM for 24, 48, and 72 h. The optimum time for the effectiveness of both drugs was found to be 72 h. The IC_50_ values of DAB in A375 and RPMI 7951 melanoma cells were 0.7 ± 0.4 µM and 0.9 ± 0.5 µM, respectively, at 72 h. The IC_50_ values of TM were 5.0 ± 1.2 µM and 7.2 ± 1.0 µM in A375 and RPMI 7951 cells, respectively.

**Fig. 1:**
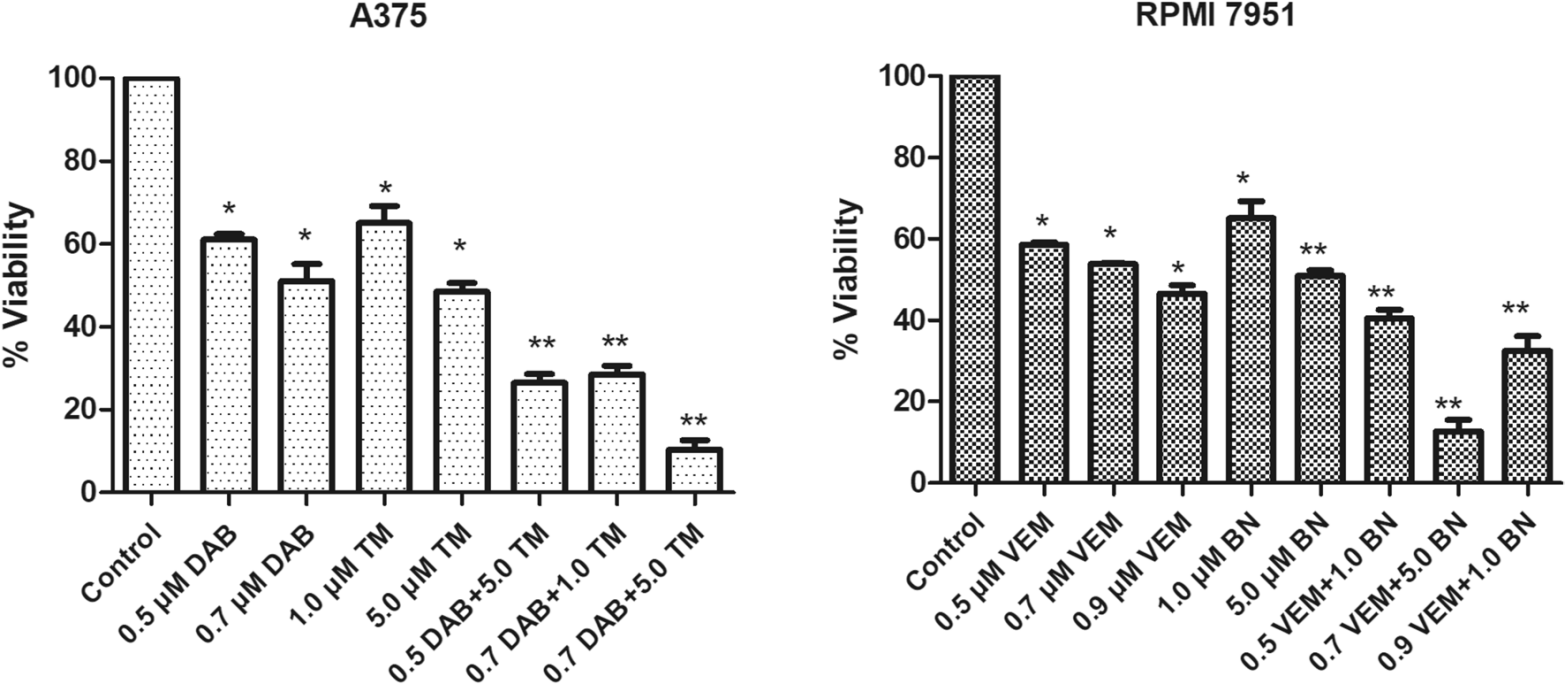
Effect of DAB, TM, and their combinations on the viability of A375 and RPMI 7951 melanoma cells. The viability of A375 and RPMI 7951 melanoma cells was assessed after 72 h of treatment with increasing concentrations of DAB, TM, and their various combinations. Cell viability was measured using MTT assay, and results are expressed as mean ± standard deviation of at least three independent experiments. A dose-dependent decrease in cell viability was observed upon treatment with DAB and TM individually, as well as in combination. The combination treatment led to a more pronounced reduction in viability compared to single-agent treatments, suggesting a potential synergistic or additive effect

Combinations at varying rates were made using the calculated IC_50_ values of each drug and applied to human melanoma cells. The synergistic combinations were evaluated using Calcusyn software via combination index (CI) values. Combination experiments showed synergistic/strong synergistic cytotoxicity in melanoma cells, A375 and RPMI 7951, at 72 h, as compared with any agent alone (Fig. 1). As shown in Fig. 1, 0.7 µM DAB and 5.0 µM TM resulted in a 48.9 and 49.1% decrease, respectively, in the viability of A375 cells, but the combination resulted in an 89.8% decrease in the viability, indicating strong synergistic cytotoxic effect (*p < 0.05). In RPMI-7951 cells, 0.7 µM DAB and 5.0 µM TM resulted in a 49.3 and 49.1% decrease in cell viability, respectively, but the combination resulted in an 87.3% decrease in the cell viability indicating a strong synergistic cytotoxic effect at 72 h (*p < 0.05). All the concentrations for each combination found to be synergistic/strong synergistic in A375 and RPMI 7951 cells are presented in Table 1. Subsequent experiments were carried out with the combination [DAB (0.7 µM) + TM (5.0 µM)] having the strongest synergistic activity in both melanoma cells at 72 h.

**Table 1:** Combination index values of DAB/TM combination in A375 and RPMI-7951 melanoma cells

A375			RPMI-7951		
DAB (0.5 µM) + TM (5.0 µM)	0.378	Synergism	DAB (0.5 µM) + TM (1.0 µM)	0.412	Synergism
DAB (0.7 µM) + TM (1.0 µM)	0.495	Synergism	DAB (0.7 µM) + TM (5.0 µM)	0.210	Strong synergism
DAB (0.7 µM) + TM (5.0 µM)	0.198	Strong synergism	DAB (0.9 µM) + TM (1.0 µM)	0.125	Strong synergism

### DAB/TM combination-induced apoptotic cell death in melanoma cells

**Fig. 2:**
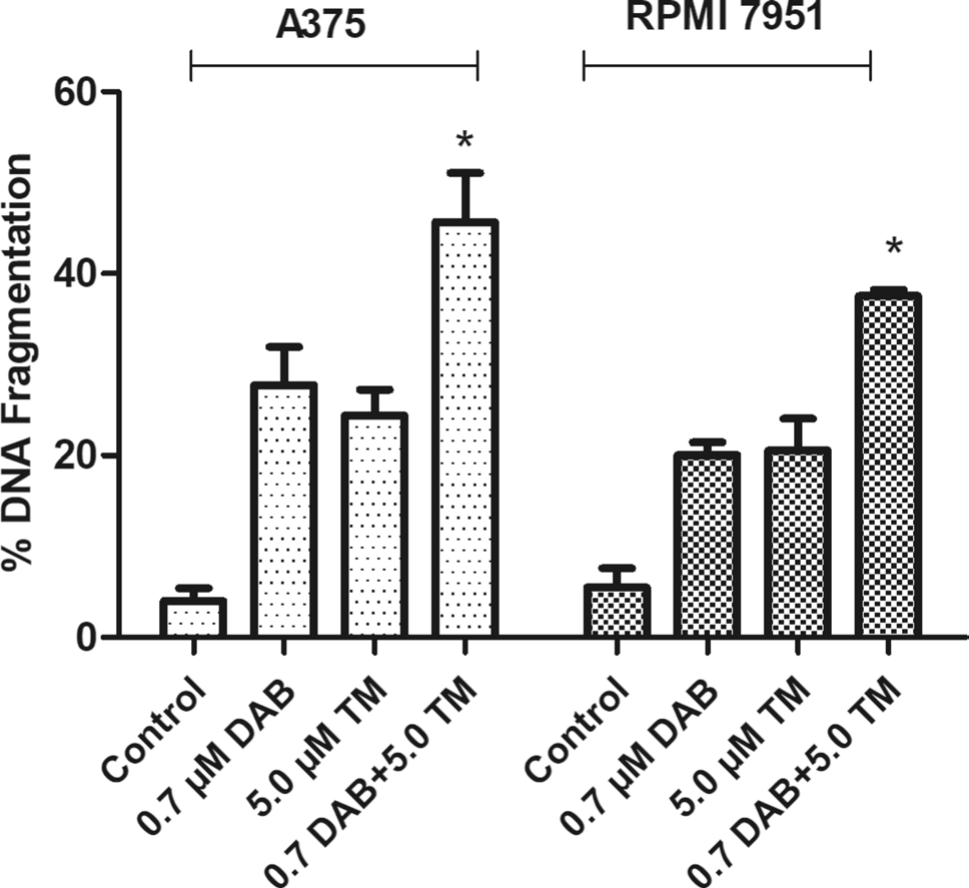
Induction of DNA fragmentation in A375 and RPMI 7951 melanoma cells. Quantification of DNA fragmentation in A375 and RPMI 7951 melanoma cells treated with 0.7 µM DAB, 5.0 µM TM, and their combination (DAB + TM) for 72 h. The extent of DNA fragmentation was assessed using ELISA and is presented as mean ± standard deviation. A statistically significant increase in DNA fragmentation was observed upon treatment with DAB, TM, and their combination compared to the control group (*p* < 0.05). The combination of DAB and TM resulted in a synergistic effect, leading to a higher level of DNA fragmentation than either treatment alone

To examine the induction of apoptotic cell death in response to DAB/TM combination treatment in A375 and RPMI 7951 melanoma cells, a DNA fragmentation assay was performed. Results showed that DAB and TM alone induced DNA fragmentation in a concentration-dependent manner in both melanoma cells (data not shown) (*p < 0.05). DAB in combination with TM also resulted in synergistic induction of DNA fragmentation in melanoma cells as compared to untreated controls and drug-alone-treated cells (Fig. 2) (*p < 0.05).

When A375 cells were exposed to 0.7 µM DAB and 5.0 μM BN, there was a 25.7%- and 24.4%-fold increase in DNA fragmentation, respectively. However, the combination induced a 45.8% increase in DNA fragmentation versus untreated controls (*p < 0.05) (Fig. 2). When RPMI 7951 was exposed to 0.7 µM DAB and 5.0 μM BN, there was a 20.8 and 20.6% increase in DNA fragmentation, and the combination induced a 39.8% increase in DNA fragmentation as compared to untreated controls and drug-alone-treated cells (Fig. 2) (*p < 0.05).

### DAB/TM combination induced mitochondria-mediated apoptosis pathway in melanoma cells

**Fig. 3:**
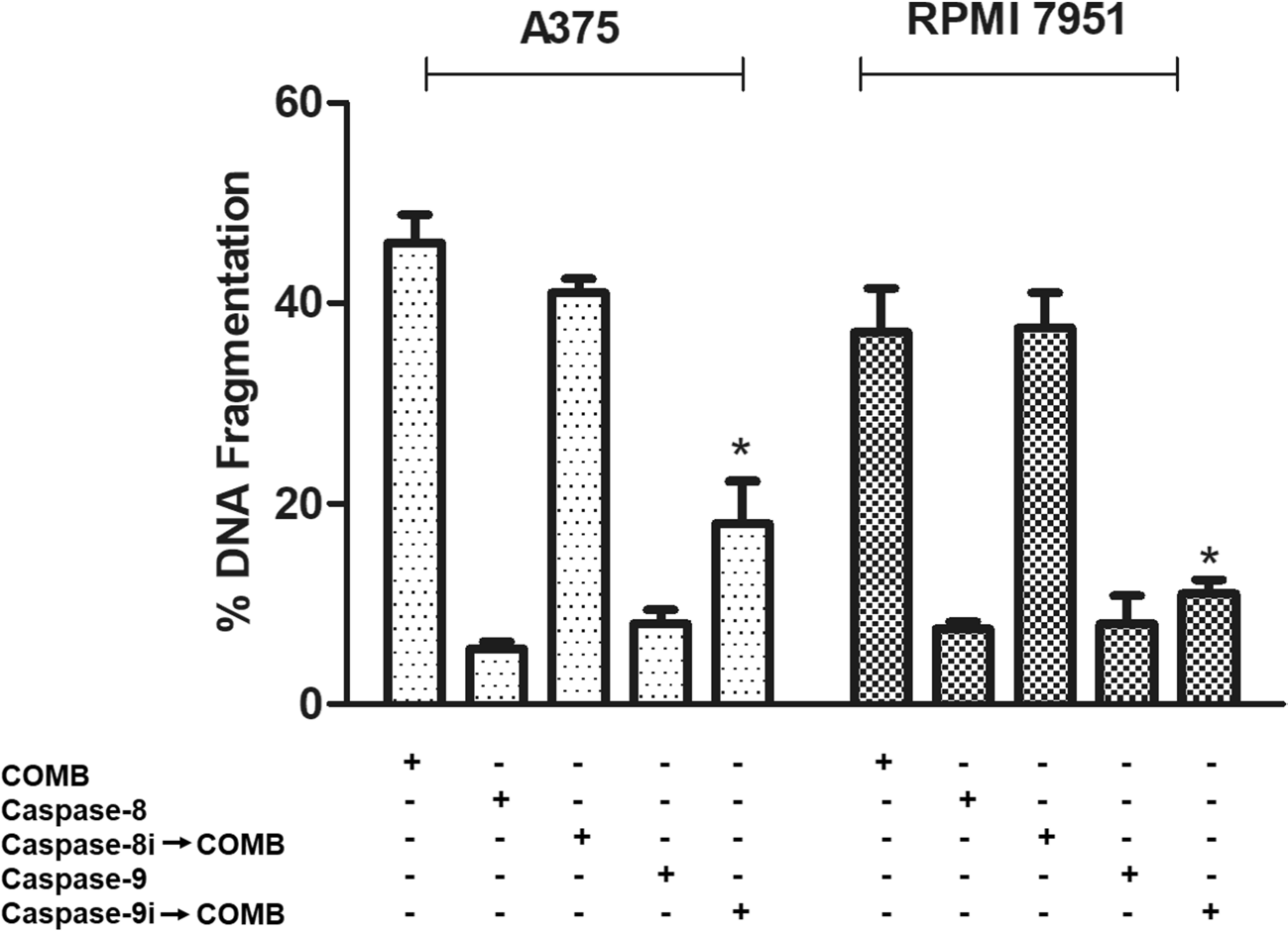
Effect of caspase-8 and caspase-9 inhibition on DNA fragmentation in A375 and RPMI 7951 melanoma cells. DNA fragmentation levels were assessed in A375 and RPMI 7951 melanoma cells after 72 h of treatment with the combination of 0.7 µM DAB and 5.0 µM TM (COMB), in the presence or absence of caspase inhibitors. Cells were pretreated with caspase-8 inhibitor (caspase-8i) and caspase-9 inhibitor (caspase-9i) to determine the involvement of extrinsic and intrinsic apoptotic pathways in COMB-induced DNA fragmentation. DNA fragmentation was quantified using ELISA results that were expressed as mean ± standard deviation. A significant reduction in DNA fragmentation was observed upon inhibition of caspase-8 and caspase-9, indicating that both extrinsic and intrinsic apoptotic pathways contribute to COMB-induced cell death (*p* < 0.05)

To investigate the cellular pathways underlying combination-induced apoptosis, and to identify which apoptotic pathways (extrinsic and/or intrinsic) are activated in melanoma cells, specific inhibitors were used. Melanoma cells were pretreated with Caspase-9 specific inhibitor (z-LEHD-fmk, Caspase-9i) and then exposed to combination treatment, and the result showed that there was a decrease in cell death as compared to combination treatment; however, pretreatment with specific Caspase-8 inhibitor (z-IETD-fmk, Caspase-8i) did not affect the combination-induced cell death (Fig. 3). These results revealed that combination treatment predominantly activates the mitochondria-mediated pathway of apoptosis in melanoma cells.

**Fig. 4:**
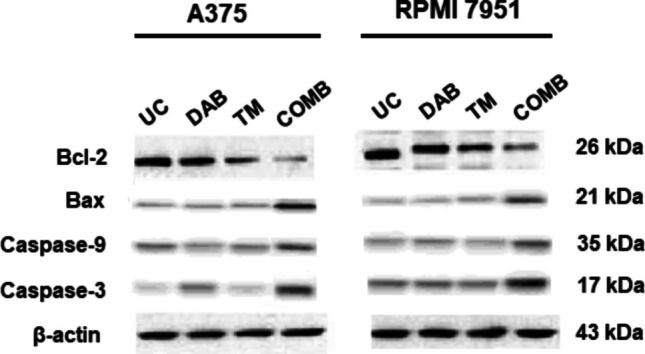
Protein expression levels of Bcl-2, Bax, caspase-9, and caspase-3 in A375 and RPMI 7951 melanoma cells after 72 h of treatment with the 0.7 µM DAB + 5.0 µM TM combination. Western blot analysis shows a decrease in the anti-apoptotic protein Bcl-2 and an increase in the pro-apoptotic protein Bax compared to the untreated control (UC). Additionally, the activation of caspase-9 and caspase-3 indicates the induction of apoptosis. β-Actin was used as a loading control

To confirm the activation of the mitochondria-mediated apoptotic pathway, the protein levels of key proteins involved in this pathway were examined after combination treatment. In both melanoma cells, Bcl-2 protein levels were reduced by 3.6-fold and the levels of Bax, caspase-9, and caspase-3 were induced by 4.6-, 3.9-, and 4.3-fold in A375 cells, respectively. In RPMI 7951 cells, Bcl-2 levels were reduced by 2.8-fold and Bax, caspase-9, and caspase-3 levels were induced by 3.2-, 3.8-, and 4.2-fold, respectively (Fig. 4).

### Effect of combination treatment on ERK1/2 and MITF expressions

**Fig. 5:**
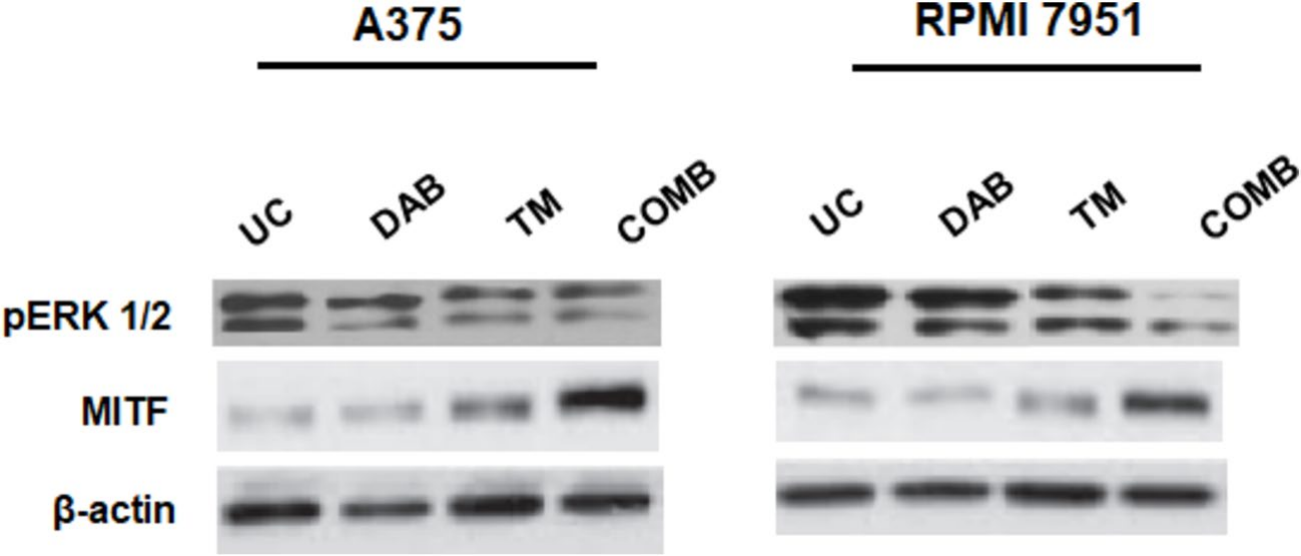
Protein levels of pERK1/2 and MITF after treatment with the 0.7 µM DAB + 5.0 µM TM combination in A375 and RPMI 7951 melanoma cells at 72 h (UC: untreated control). Western blot analysis demonstrates significant changes in protein expression compared to the untreated control (UC). β-Actin was used as a loading control

To identify the possible action mechanism of combination treatment, levels of pERK1/2 and MITF were investigated via western blot analysis. As shown in Fig. 5, levels of pERK1/2 were reduced by 2.7-fold and MITF levels were induced by 3.1-fold in A375 cells. Similarly, in RPMI 7951 cells, pERK1/2 levels were reduced by 3.4-fold and MITF levels were induced by 2.8-fold by the combination treatment as compared to single drugs or untreated control (*p < 0.05).

### Let-7a regulates caspase-3-dependent apoptotic cell death of DAB/TM-treated melanoma cells

**Fig. 6:**
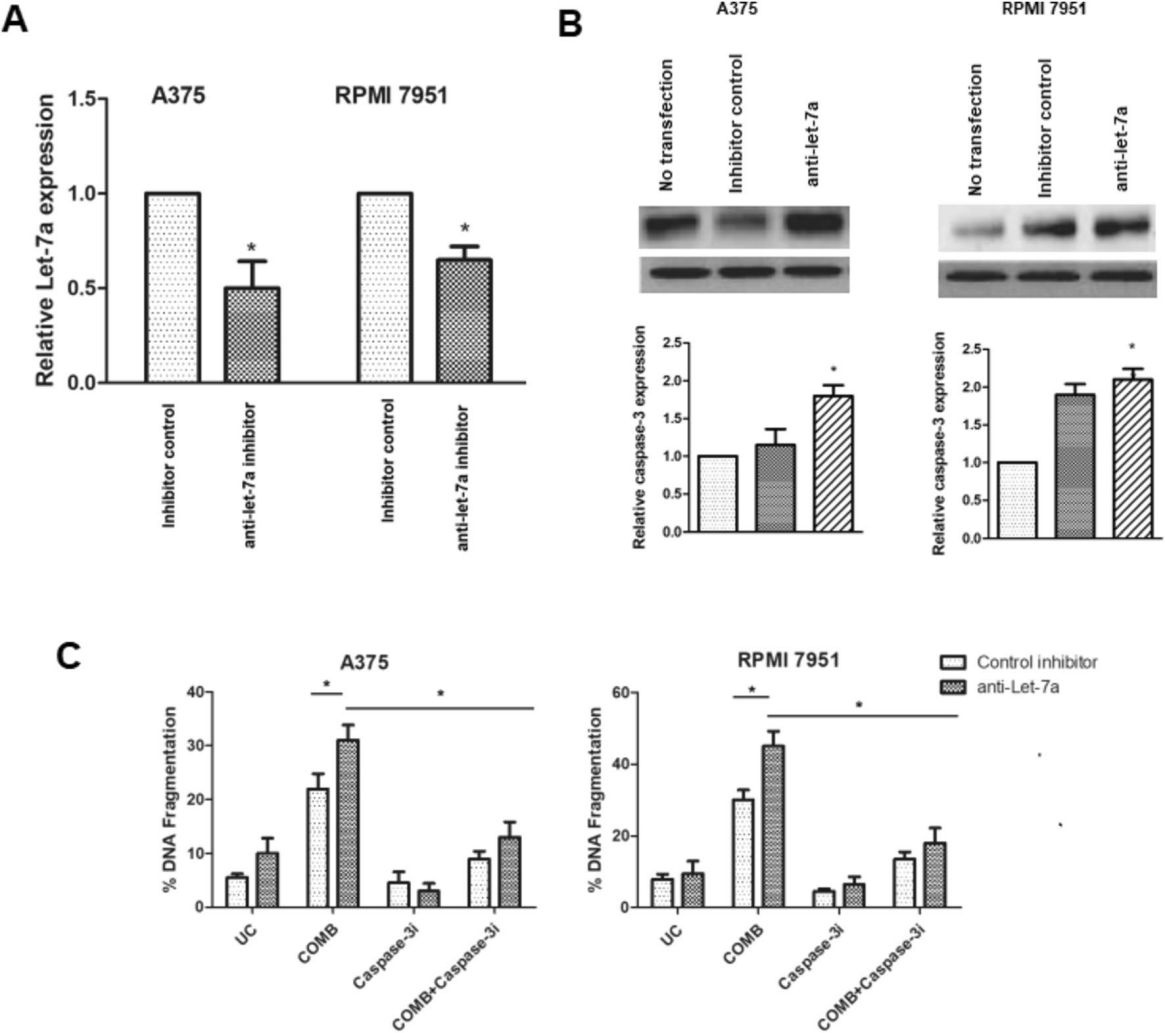
The effect of let-7a on caspase-3 expression and combination-induced apoptosis in A375 and RPMI 7951 cells. **A** Relative Let-7a levels in melanoma cells measured by quantitative real-time PCR (*p < 0.05). **B** Caspase-3 levels in melanoma cells measured by Western blot analysis. The calculation of relative caspase-3 expression in transfected cells was performed by comparing it to the expression level in non-transfected cells (*p < 0.05). **C** Combination-induced apoptosis in melanoma cells as measured by DNA fragmentation assay. After transfection with anti-let-7a inhibitor, the cells were exposed to combination treatment with or without 25 µM of caspase-3 inhibitor for 72 h (*p < 0.05)

We investigated the role of let-7a in the combination-induced apoptosis in both A375 and RPMI 7951 cells. Ectopic let-7a expression decreased the extent of combination-induced DNA fragmentation in both melanoma cells as compared to the control transfection and no transfection (data not shown). To confirm that the drug-induced apoptosis attenuated by let-7a is through the caspase-3, the effect of caspase-3 inhibitor on combination-induced apoptosis in melanoma cells upon ectopic anti-let-7a inhibitor transfection was examined. Knockdown of let-7a by transfection increased the caspase-3 level (Fig. 6) and also slightly the apoptotic cell population in melanoma cells. Moreover, the let-7a knockdown also enhanced the combination-induced apoptosis in cells while the enhancement effect was counteracted by the caspase-3 inhibitor (Fig. 6). These results support the involvement of caspase-3 in let-7a regulation of apoptosis in melanoma cells.

## Discussion

The development of resistance through BRAF-inhibitor bypass and the emergence of RAS-driven secondary cancers in response to BRAF inhibition has necessitated the exploration of combination therapies involving both BRAF and MEK inhibitors. Combination therapies involving BRAF and MEK inhibitors have become the standard of care for advanced melanoma with BRAF mutations. The trametinib/dabrafenib and cobimetinib/vemurafenib combinations were approved by the FDA in 2014 and 2015, respectively [18, 19]. While these combinations have shown promise in prolonging responses and reducing the incidence of RAS-driven secondary malignancies, toxic effects and acquired drug resistance remain the biggest challenges.

Here, the mechanism of synergistic effects of DAB and TM was investigated on drug-resistant A375 and RPMI 7951 melanoma cells. DAB (0.5, 0.7, and 0.9 µM) and TM (1.0, 5.0 and 7.2 µM) at subtoxic doses were combined. All tested combinations were found to have synergistic or strong synergistic cytotoxic effects in melanoma cells. Among them, the combination of 0.7 µM DAB and 5.0 µM TM showed synergistic cytotoxic effects on both melanoma cells, so this combination was chosen for further studies.

To determine the intracellular effects of the combination in melanoma cells, we investigated the effects of combination treatment on the ERK1/2 signaling pathway and subsequently on MITF, which is critical for melanoma cells. It has been shown that the BRAF V600E mutation leads to hyperactivation of ERK and promotes proliferation in melanocytes. Ectopic expression of BRAFV600E in human melanocytes has been shown to decrease the levels of MITF protein [20]. The role of this downregulation in proliferation is not entirely clear and it is not yet known whether it is a consequence of ERK activation or a requirement for proliferation. However, in murine BRAF-transformed melanocytes, overexpression of MITF inhibits proliferation, indicating that the regulation of MITF expression levels by the MAPK pathway plays an important role in the growth of BRAF-driven melanoma. Combination treatment inhibited ERK1/2 protein levels and induced MITF protein levels in A375 and RPMI 7951 cells as compared to single drugs and untreated controls.

One of the resistance mechanisms to BRAF/MEK inhibitors involves the up-regulation of pro-survival factors, which enable melanoma cells to evade apoptosis even when ERK is completely or efficiently inhibited [21]. Therefore, we investigated apoptotic events in melanoma cells after combination therapy. The possible synergistic apoptotic effect of the DAB/TM combination in melanoma cells was evaluated by measuring the DNA fragmentation levels. DNA fragmentation levels were significantly induced in combination-treated A375 and RPMI 7951 melanoma cells as compared to single drugs and untreated control. To determine which apoptotic pathway was triggered by combination therapy, specific caspase-8 and caspase-9 inhibitors were used. The extrinsic and intrinsic pathways are two major pathways that can lead to apoptosis. The extrinsic pathway is initiated by the binding of extracellular signals such as death ligands (e.g., FasL, TNFα) to their corresponding receptors on the cell surface. The binding of the ligands to the death receptors triggers the recruitment of the adaptor protein Fas-associated death domain protein (FADD) and pro-caspase-8 to the death-inducing signaling complex (DISC). The recruitment of caspase-8 to the DISC leads to its activation, which can directly cleave and activate effector caspases (e.g., caspase-3, −6, and −7) or interact Bcl-2 family members to induce apoptosis [22]. The intrinsic (mitochondria-mediated) pathway, on the other hand, is initiated by intracellular stress signals. These signals cause the release of cytochrome *c* from the mitochondria into the cytosol. Once released into the cytoplasm, cytochrome *c* binds to apoptotic protease activating factor 1, leading to the formation of the apoptosome complex. The apoptosome then activates caspase-9, which in turn activates downstream effector caspases [23]. Experiments with specific inhibitors revealed that the combination of DAB with TM induced the mitochondria-mediated apoptotic pathway in both melanoma cells. After detecting that the intrinsic apoptotic pathway was induced by the combination treatment, the expression levels of some pro- and anti-apoptotic molecules involved in this pathway were investigated by the Western blot analysis to confirm that this pathway was activated. As a result of the combination treatment, Bcl-2, an anti-apoptotic protein, decreased in both melanoma cell lines, while a significant increase was observed in pro-apoptotic Bax and pro-apoptotic caspase-9 and caspase-3 protein levels.

Furthermore, we investigated the role of let-7a in the combination-induced apoptosis in melanoma cells. Let-7a has been implicated in the regulation of various cancers beyond melanoma, influencing key oncogenic pathways involved in proliferation, apoptosis, and metastasis. In lung cancer, Let-7a has been shown to suppress RAS signaling, thereby inhibiting tumor progression and enhancing sensitivity to targeted therapies. Similarly, in breast cancer, Let-7a downregulates HMGA2 and c-Myc, contributing to reduced cell proliferation and increased apoptotic activity. Studies in colorectal and pancreatic cancers further suggest that Let-7a modulates cell cycle regulators and epithelial-mesenchymal transition (EMT)–associated genes, preventing tumor invasion and metastasis. Given its broad regulatory role in multiple malignancies, Let-7a represents a promising therapeutic target, with potential applications in personalized medicine approaches aimed at restoring its expression or enhancing its tumor-suppressive effects [24, 25]. This is supported by the overexpression of let-7a, which reduces the susceptibility of cells to combination-induced apoptosis.

In this study, it has been confirmed that Let-7a is a crucial regulator of the sensitivity of melanoma cells to combined therapy-induced apoptosis by targeting caspase-3, which is considered the primary executor of caspase of apoptosis. While our findings provide valuable insights into the synergistic effects of dabrafenib (DAB) and trametinib (TM) in drug-resistant melanoma cells, certain limitations should be acknowledged. First, the study is based solely on in vitro experiments, and the absence of in vivo validation restricts the translational applicability of the results. Future studies incorporating animal models could further substantiate the clinical relevance of Let-7a-mediated apoptosis in melanoma. Second, while we demonstrated that the combination treatment induces the mitochondria-mediated apoptotic pathway, the precise upstream regulators and additional signaling cascades contributing to this effect remain to be elucidated. Other microRNAs or compensatory survival mechanisms may play a role in the observed cytotoxicity. Despite these limitations, our study provides a foundation for understanding the mechanistic role of Let-7a in melanoma apoptosis and highlights its potential as a therapeutic target for overcoming drug resistance.

## Data Availability

The data supporting the findings of this study are available from the corresponding author upon reasonable request.
